# Rare metastasis in a patient with BRAF-mutated rectal cancer: choroidal metastasis – case report and literature review

**DOI:** 10.3205/oc000235

**Published:** 2024-04-19

**Authors:** Hacer Demir, Mustafa Muhterem Ekim, Esra Özgül, Sena Ece Davarci, Meltem Baykara

**Affiliations:** 1 Department of Medical Oncology, Afyonkarahisar University of Health and Sciences, Afyonkarahisar, Turkey; 2 Department of Ophthalmology, Park Hayat Hospital, Afyonkarahisar, Turkey; 3 Department of Radiology, Afyonkarahisar University of Health and Sciences, Afyonkarahisar, Turkey

**Keywords:** BRAF mutation, choroid metastasis, rectal cancer

## Abstract

**Purpose:**

Colorectal cancers are common and have high mortality, and metastasis is common in follow up. Choroidal metastasis is encountered rarely in rectum cancers, and there is no previous case reported from Turkey. We present our patient who developed choroidal metastasis in his cancer follow-up.

**Case report:**

A 74-year-old male patient had undergone operation due to the diagnosis of rectum cancer two years ago, and lung (L) metastasis developed in the 4^th^ month after the adjuvant therapy, but he refused to receive treatment and remained out of follow-up. The patient presented with complaints of decreased vision and light flashes in his eye 21 months after the diagnosis.

**Management and outcome:**

Ocular examination revealed a choroidal mass and radiologically choroidal and multiple brain metastases were detected. In our case, whole-brain radiotherapy was administered in the treatment since there were also multiple brain metastases. However, as the ECOG (Eastern Cooperative Oncology Group) performance status of the patient was 3–4 after radiotherapy, systemic treatment was not considered appropriate, and the best supportive care was given. The patient died 2 months after the diagnosis of choroidal metastasis.

**Conclusion:**

Currently, there are few suggestions in case reports regarding appropriate treatment approaches for the treatment of rectal cancerchoroidal metastases. Multidisciplinary approaches may be effective for local and systemic treatment. Our case highlights a pathological entity with poor prognosis, which is rarely encountered during the course of rectal adenocarcinomas, and it is the first case of choroidal metastasis reported from our country. However, we believe that it will be important to draw attention to the fact that it is the first reported case of choroid metastasis in a rectal cancer patient with a BRAF V600 E mutation, and patients with BRAF V600 E mutation may develop metastasis to atypical areas due to their aggressive biology.

## Introduction

Incidence of colorectal cancer (CRC) and mortality have been decreasing worldwide, but it still remains the leading cause of morbidity. According to data of the World Health Organization, CRC is the third most common cancer in males and second in females, and it is the third most common cause of cancer deaths in the USA for both women and men [1].

Metastasis of colorectal cancer is common. While 20% of the patients are in the metastatic stage at the time of diagnosis, nearly 30% of the patients develop metastasis during their follow-up.

The most common regions of metastasis are liver (77%), peritoneum (25%), and lungs (22%) [2], [3].

Metastatic tumors of the intraocular region are more common than primary ones, and the most common metastatic site is the choroidal site presumably due to its rich vasculature, constituting 80% of the cases [4], [5]. It is frequently seen during the course of breast and lung adenocarcinomas, and non-adenocarcinoma histology is rare. While metastasis in the ocular region is less common in colorectal cancers, 4% of the cases are caused by colorectal adenocarcinoma [5], [6].

Metastases can be seen in the form of unilateral or bilateral involvement; usually lung and colon cancer metastases are seen at an early period, while breast cancer metastasis develops in the later period. Patients with choroidal metastases have a poor prognosis and 3-year survival is around 14% in metastases due to colorectal cancer [7].

Although there is no general consensus on the management and treatment of patients with choroidal metastasis, radiotherapy, chemotherapy and intravitreal bevacizumab treatments have been used. Here, we report a case of a patient with rectal cancer who presented with the complaint of decreased vision 23 months after diagnosis and who was diagnosed with choroidal metastasis.

## Case description – management outcome

A 70-year-old male patient was admitted to the hospital in December 2018 with a complaint of hematochezia for 2 months. Upon detecting an ulcerovegetan mass in the rectum in colonoscopy, the patient, who underwent low anterior resection operation with a diagnosis of rectal cancer, applied to the medical oncology outpatient clinic for treatment. As a result of the pathology, moderately differentiated adenocarcinoma, the tumor invaded the serosa, lymphovascular invasion was present, and tumor was found in 11 of the 12 lymph nodes removed. Vertical and horizontal surgical margins were noted as negative. At the time of admission, the patient’s staging was performed first, and determined as T3N2M0 according to American Joint Committee on Cancer (AJCC) staging system; postoperative adjuvant radiotherapy with simultaneous capecitabine and capecitabine/oxaliplatin-based systemic chemotherapy treatments were applied for 6 months, and he was followed up after treatment.

Radiological imaging performed 4 months after the end of adjuvant therapy revealed multiple lung metastases, the largest of which was 27x23 mm, and the patient was restaged as stage 4 metastatic rectal cancer. When the RAS wild-type BRAF V600E mutation was observed in the KRAS/NRAS/BRAF analysis, 5-fluorouracil, leucovorin, irinotecan and anti-EGFR (epidermal growth factor receptor) monoclonal treatment was initiated for the patient. The patient, who had been treated for 2 months, was re-evaluated after another palliative surgery upon the development of ileus, and in positron emission tomography and computed tomography (PET/CT), progression in lung metastases and new metastatic nodules in the peritoneum were detected. However, the patient refused to receive treatment and did not continue his follow-ups for 8 months.

**Figure 1:**
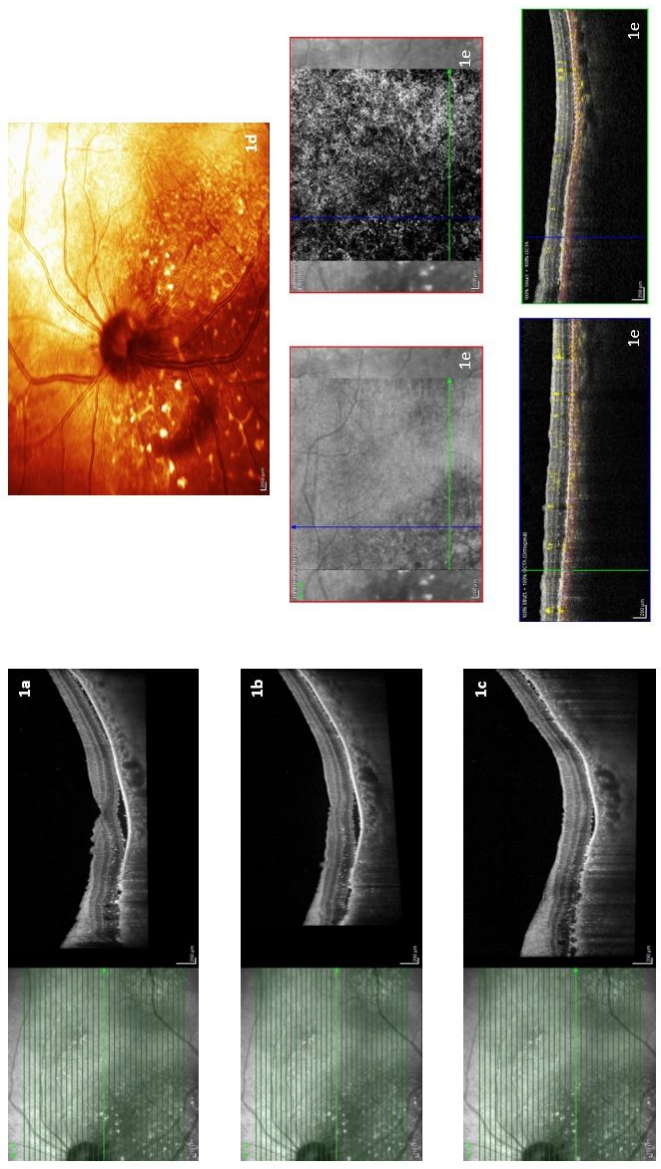
Fundus examination. (a) OCT showing retinal detachment under the macula, subretinal fluid in the temporal area, and diffuse hyperreflective foci in the retina, (b) increase in choroidal thickness (>500 µ), (c) a low reflective mass originating from under the optic disc in the lower nasal quadrant, causing shadowing, and effacement of the choroidal capillaries, (d) infrared imaging showing hyperautofluorescent yellowish mottled lesions under the optic disc at the posterior pole, and (e) OCTA images with decreased choroidal capillaries in the lower nasal quadrant

Upon restaging examinations performed when he presented with diffuse ascites in the abdomen in August 2020, progression in lung cancer metastases and new metastatic lesions in the adrenal and bone were detected, and it was again evaluated as a progressive disease. First, radiation therapy was applied to vertebral metastases at risk of compression fracture, and then zoledronic acid treatment was planned. After radiotherapy, the patient started complaining of loss of vision in the left eye, blurred vision, and myodesopsia in September 2020. In the ophthalmic examination, best-corrected visual acuity was 10/10 in the right eye, and 4/10 in the left eye. Intraocular pressure of both eyes was 12 mmHg. Subretinal fluid (serous retinal detachment) in the lower half of the retina, including the macula, and choroidal hyperpigmented lesion (metastasis?) in the lower temporal area, and hyperreflective dense tissue infiltration in the lower half of the optic disc, disrupting the choroidal vasculature, were observed (fundus examination image is shown Figure 1a–e (Fig. 1)).

**Figure 2:**
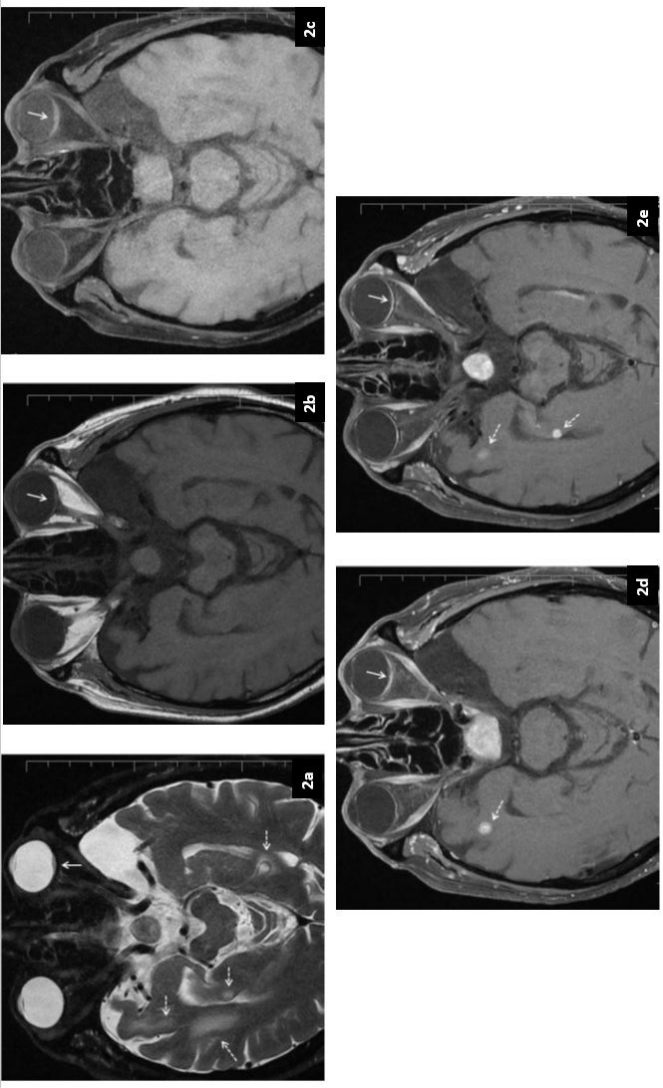
Axial fat-saturated T2 (a), axial T1 (b), and axial fat-saturated T1 (c) weighted images demonstrate diffuse choroidal thickening in the posterior part of the left orbital globe (arrows). Axial contrast enhanced fat-saturated T1 weighted (d, e) images show contrast enhancement of thickened left posterior choroidal layer (arrows). Also metastatic lesions in the brain parenchyma and vasogenic edema around them are seen in all images (dashed arrows).

In brain and orbit magnetic resonance imaging (MRI), choroidal thickening and contrast enhancement in the posterior part of the left orbital globe was seen on MRI images found compatible with choroidal metastasis. In addition, metastatic lesions in the brain parenchyma and vasogenic edema around them were also detected (Figure 2 (Fig. 2)). There upon, 3,000 cGy radiotherapy was administered to the patient’s whole brain in 10 fractions in September 2020. However, it was decided to give the best supportive care to the patient whose performance deteriorated after radiotherapy. The patient died on November 16, 2020. Progression-free survival (PFS) was determined as 23 months from the diagnosis.

## Discussion

The present case illustrates a rare site of metastasis for rectal cancer. Among ocular malignancies, metastatic tumors are encountered more often than primary tumors. They can be observed frequently during the course of breast and lung cancers, and they accompany gastrointestinal cancers in 4% of cases [8]. Although colorectal cancers are very common worldwide and rank third among the cancer-related deaths, their ocular metastasis rate is very low; so far, this metastasis has been seen in 25 patients in the light of our literature review, and there are no cases reported in the Turkish population [9], [10], [11].

In our research, which we carried out using the keywords “choroidal metastasis”, “cancer” and “colorectal” in PubMed until 2020, 22 cases were identified in patients with colorectal cancer in addition to our current patient who developed choroidal metastasis, 12 of them were patients with only the diagnosis of rectum cancer. In 19 of these patients, ocular metastasis was only in the form of choroidal involvement, while in 3 cases there was additional optic nerve or retinal involvement.

Reviewing the primary tumor sites of patients who developed choroidal metastasis in the literature, it was seen that it originated from the colon in 12 patients, the rectum in 12 patients, and the colorectal region in 1 patient. In most of the case reports, the TNM (tumor, node, metastasis) stage of the patients was not specified [4], [10], [11], [12], [13], [14], [15], [16], [17], [18], [19]. Cases with rectum cancer reported in the literature are summarized in Table 1 (Tab. 1). The information on cases for which the full texts of the case reports could not be accessed was obtained from other literature.

The mean age of 12 patients who developed choroidal metastases with the primary tumor site in the rectum was 53.7 years. The mean time that elapsed from the diagnosis of primary tumors in patients until the development of choroidal metastasis was 28.5 months (ranging from the time of diagnosis up to 96 months). Only 3 patients had synchronous choroidal metastasis at the time of diagnosis [12], [14], [15]. In addition to choroid metastasis, patients also had lung, brain, bone, and liver metastases. The primary tumor of our patient also originated from rectum and choroidal metastasis developed at 22 months after diagnosis, and his age was 72 years when choroidal metastasis was detected. Along with choroidal metastasis, the patient also had multiple central nervous system metastases, lung, bone, adrenal and peritoneal metastases.

In cases reported in the literature, the most common symptom that is the cause of ophthalmological examination is vision loss, and additionally, some patients presented with the complaints of blurred vision, pain in the eye and flashes of light. In our patient’s ophthalmological examination upon the complaint of decreased vision in his left eye, it was found that the best-corrected visual acuity in the left eye was 4/10, and intraocular pressures were normal in both eyes. Fundus examination revealed subretinal fluid in the lower half of the retina, including the macula; choroidal metastasis was detected in the lower half of the optic disc, in the choroidal layer, with hyperreflective tissue infiltration disrupting the choroidal vasculature.

While enucleation was performed in 3 of the patients reported in the literature, enucleation is currently limited to those with severe eye pain and uncontrolled glaucoma in advanced-stage patients with diffuse ocular involvement [10], [14], [15].

Whole cranial radiotherapy was administered in the treatment due to the fact that our patient also had multiple brain metastases along with choroidal metastasis.

It is stated that survival was between 1 and 31 months after the diagnosis of choroidal metastasis in patients who had rectal cancer and developed choroidal metastasis [10]. In the study where patients developing choroidal metastases together with colorectal cancer were evaluated, Tei et al. reported the median survival as 5.6 months [9]. In our case, survival was 2 months after the diagnosis of choroidal metastasis. In the case of choroidal metastasis development in patients with colorectal cancer, the prognosis is poor, and other accompanying organ metastases should also be investigated in these patients.

The KRAS, NRAS and BRAF states of the cases are not mentioned in the literature. In the molecular analysis examined in our case, KRAS, NRAS were identified as wild type but BRAF V600E as mutant. The BRAF mutation is found in 7–12% of all colorectal cancers, and patients with the mutation have more aggressive tumor biology and resistance to standard chemotherapy, so the prognosis in these patients is poor [20], [21]. While the median overall survival (OS) is 12 months in patients with BRAF mutation, that for those with wild type is around 30 months. In our case survival was 24 months even though the patient had BRAF mutation and did not receive effective treatment in the metastatic stage. The clinical picture of our patient suggests that due to the aggressive biology of BRAF mutant patients, atypical site metastases such as choroidal metastasis may occur.

As choroidal colorectal metastases are not common, data on optimal treatment of these patients is limited and limited only to non-comparative case reports involving various treatment approaches. Treatment in symptomatic patients depends on the patient’s performance status and the extent of spread of metastatic disease. Currently, applications of systemic chemotherapy, radiation therapy, chemoradiotherapy and intravitreal bevacizumab treatments have been reported in relation to the treatment of choroidal metastasis [11], [12], [13], [14], [15], [18], [19], [22]. In patients with vision loss and severe pain due to uncontrolled glaucoma, enucleation is also among the treatment options [10], [14], [15]. In the article published by Neto et al. in early 2020, it was reported that local photodynamic treatment might also be effective in addition to the systemic treatment in a case with choroidal metastasis of colon cancer [23].

## Conclusion

In colorectal cancers, choroidal metastases are quite rare, and the present case is the first one reported from Turkey to the best of our knowledge. In our case, whole-brain radiotherapy was administered in the treatment since there were also multiple brain metastases. However, as the ECOG performance status of the patient was 3–4 after radiotherapy, systemic treatment was not considered appropriate, and the best supportive care was given. The patient died 2 months after the diagnosis of choroidal metastasis.

Clinicians should also consider the possibility of ocular metastasis if patients with advanced colorectal cancer have vision problems and especially in the follow-up of BRAF mutant patients, attention should be paid to the development of atypical metastasis.

In conclusion, further experience and long-term follow-up are required for the optimal treatment of colorectal cancer choroidal metastases.

## Notes

### Author’s ORCID

Sena Ece Davarci, MD: 0000-0003-1142-9411

### Statement of human and animal rights

All procedures performed in this study were in accordance with the ethical standards of the institutional and/or national research committee and with the 1964 Helsinki declaration and its later amendments or comparable ethical standards. No animal or human studies were carried out by the authors for this article.

### Scientific responsibility statement

The authors declare that they are responsible for the article’s scientific content including study design, data collection, analysis and interpretation, writing, and all of the preparation and scientific review of the contents and approval of the final version of the article.

### Competing interests

The authors declare that they have no competing interests.

## Figures and Tables

**Table 1:**
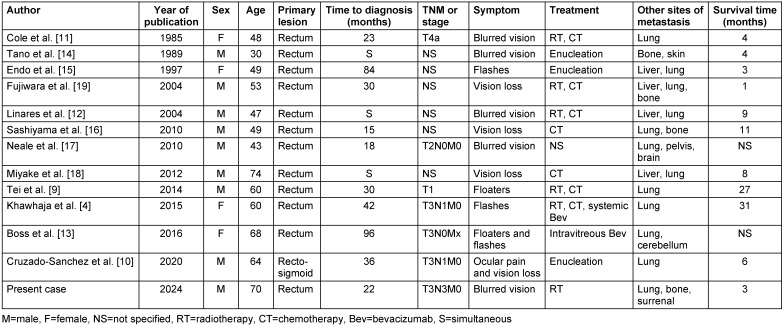
Choroid metastasis from rectal adenocarcinoma (table adapted from [10])

## References

[1] Siegel RL, Miller KD, Jemal A (2019). Cancer statistics, 2019. CA Cancer J Clin.

[2] Hess KR, Varadhachary GR, Taylor SH, Wei W, Raber MN, Lenzi R, Abbruzzese JL (2006). Metastatic patterns in adenocarcinoma. Cancer.

[3] Qiu M, Hu J, Yang D, Cosgrove DP, Xu R (2015). Pattern of distant metastases in colorectal cancer: a SEER based study. Oncotarget.

[4] Khawaja MR, Minturn JT, Spittler AJ, Chiorean EG (2015). Ocular metastasis of colorectal cancer: An uncommon presentation of a common malignancy. Hematol Oncol Stem Cell Ther.

[5] Shields CL, Shields JA, Gross NE, Schwartz GP, Lally SE (1997). Survey of 520 eyes with uveal metastases. Ophthalmology.

[6] Ferry AP, Font RL (1974). Carcinoma metastatic to the eye and orbit. I. A clinicopathologic study of 227 cases. Arch Ophthalmol.

[7] Shields CL, Welch RJ, Malik K, Acaba-Berrocal LA, Selzer EB, Newman JH, Mayro EL, Constantinescu AB, Spencer MA, McGarrey MP, Knapp AN, Graf AE, Altman AJ, Considine SP, Shields JA (2018). Uveal Metastasis: Clinical Features and Survival Outcome of 2214 Tumors in 1111 Patients Based on Primary Tumor Origin. Middle East Afr J Ophthalmol.

[8] Chong JT, Mick A (2005). Choroidal metastasis: case reports and review of the literature. Optometry.

[9] Tei M, Wakasugi M, Akamatsu H (2014). Choroidal metastasis from early rectal cancer: Case report and literature review. Int J Surg Case Rep.

[10] Cruzado-Sanchez D, Saavedra-Mejia LA, Tellez WA, Maquera-Torres G, Serpa-Frias S (2020). Metastatic Intraocular Tumor Due to Colorectal Adenocarcinoma: Case Report and Literature Review. J Ophthalmic Vis Res.

[11] Cole MD, Farah NB (1985). The choroid--an unusual site for metastasis in patients with adenocarcinoma of the rectum – a case report. Eur J Surg Oncol.

[12] Linares P, Castanon C, Vivas S, Diz P, Garcia-Palomo A, Llano C, Olcoz JL (2004). Bilateral choroidal metastasis as the initial manifestation of a rectal cancer. J Gastroenterol Hepatol.

[13] Boss JD, Lieu P, Tewari A (2016). Effect of treatment of rectal cancer metastasis with intravitreal bevacizumab (Avastin) in patient with subretinal fluid and macular oedema: short-term follow-up. BMJ Case Rep.

[14] Tano S, Hayashi H, Momoeda S (1989). Metastasis of rectal carcinoma to the choroid: a case report. Nihon Ganka Kiyo.

[15] Endo H, Tajika T, Takebayashi H, Shiota H, Yoshida M, Kudo E (1997). A case report of choroidal metastasis from rectal cancer. Ganka rinsho iho.

[16] Sashiyama H, Abe Y, Sasagawa S, Hanada H, Hatori Y, Kubota M, Sakao S, Hiroshima K (2010). A case of choroidal metastasis from rectal cancer manifesting visual loss as the initial recurrence symptom. Jpn J Gastroenterol Surg.

[17] Neale JA, Valsdottir E, Zeger E, Shields C, Marks J (2010). Cerebral and choroidal metastases with retinal detachment, secondary to rectal cancer: a case report. World J Colorectal Surg.

[18] Miyake E, Moriwaki M, Sunada T, Takemura J (2012). Regression of choroidal metastasis from rectal cancer following chemotherapy. Atarashii Ganka.

[19] Fujiwara T, Machida S, Murai K, Tazawa Y, Baba Y, Shimooki O (2004). A case of choroidal tumor metastasized from rectal cancer. Ganka.

[20] Cohen R, Cervera P, Svrcek M, Pellat A, Dreyer C, de Gramont A, André T (2017). BRAF-Mutated Colorectal Cancer: What Is the Optimal Strategy for Treatment?. Curr Treat Options Oncol.

[21] Bernabe-Ramirez C, Patel R, Chahal J, Saif MW (2020). Treatment options in BRAF-mutant metastatic colorectal cancer. Anticancer Drugs.

[22] Lin CJ, Li KH, Hwang JF, Chen SN (2010). The effect of intravitreal bevacizumab treatment on choroidal metastasis of colon adenocarcinoma – case report. Eye (Lond).

[23] Biccas Neto L, Pulido JZ, Melo GB, Lima LH, Rodrigues EB (2020). Photodynamic Therapy of Presumed Choroidal Metastasis Secondary to Colorectal Carcinoma: Literature Review. Case Rep Ophthalmol Med.

